# Sex, age, type of diabetes and incidence of atrial fibrillation in patients with diabetes mellitus: a nationwide analysis

**DOI:** 10.1186/s12933-021-01216-7

**Published:** 2021-01-22

**Authors:** Arnaud Bisson, Alexandre Bodin, Grégoire Fauchier, Julien Herbert, Denis Angoulvant, Pierre Henri Ducluzeau, Gregory Y. H. Lip, Laurent Fauchier

**Affiliations:** 1 Service de Cardiologie, Centre Hospitalier Universitaire Et Faculté de Médecine, Université de Tours, Hôpital Trousseau, 37044 Tours, France; 2 Service de Médecine Interne, Unité D’Endocrinologie Diabétologie Et Nutrition, Centre Hospitalier Universitaire Et Faculté de Médecine, Université de Tours, Tours, France; 3 Service D’information Médicale, D’épidémiologie Et D’économie de La Santé, Centre Hospitalier Universitaire Et Faculté de Médecine, Université de Tours, EA7505 Tours, France; 4 EA4245 T2i, Université de Tours, Tours, France; 5 INRAE (Institut National de Recherche Pour L’Agriculture, l’Alimentation Et L’Environnement), Unité Mixte de Recherche Physiologie de La Reproduction Et Des Comportements, 37380 Nouzilly, France; 6 Liverpool Centre for Cardiovascular Science, University of Liverpool and Liverpool Heart & Chest Hospital, Liverpool, UK; 7 Department of Clinical Medicine, Aalborg University, Aalborg, Denmark

**Keywords:** Diabetes, Atrial fibrillation, Sex-difference

## Abstract

**Background:**

There remain uncertainties regarding diabetes mellitus and the incidence of atrial fibrillation (AF), in relation to type of diabetes, and the interactions with sex and age. We investigated whether diabetes confers higher relative rates of AF in women compared to men, and whether these sex-differences depend on type of diabetes and age.

**Methods:**

All patients aged ≥ 18 seen in French hospitals in 2013 with at least 5 years of follow-up without a history of AF were identified and categorized by their diabetes status. We calculated overall and age-dependent incidence rates, hazard ratios, and women-to-men ratios for incidence of AF in patients with type 1 and type 2 diabetes (compared to no diabetes).

**Results:**

In 2,921,407 patients with no history of AF (55% women), 45,389 had prevalent type 1 diabetes and 345,499 had prevalent type 2 diabetes. The incidence rates (IRs) of AF were higher in type 1 or type 2 diabetic patients than in non-diabetics, and increased with advancing age. Among individuals with diabetes, the absolute rate of AF was higher in men than in women. When comparing individuals with and without diabetes, women had a higher adjusted hazard ratio (HR) of AF than men: adjusted HR 1.32 (95% confidence interval 1.27–1.37) in women vs. 1.12(1.08–1.16) in men for type 1 diabetes, adjusted HR 1.17(1.16–1.19) in women vs. 1.10(1.09–1.12) in men for type 2 diabetes.

**Conclusion:**

Although men have higher absolute rates for incidence of AF, the relative rates of incident AF associated with diabetes are higher in women than in men for both type 1 and type 2 diabetes.

## Introduction

Atrial fibrillation (AF) and diabetes mellitus are common worldwide and their incidence is increasing, representing a significant public health and economic burden as well as an increase in individual risk of morbidity and mortality. Both conditions are closely related, and AF patients with diabetes are at higher risk of cardiovascular events compared to non-AF patients. Patients with diabetes are at increased risk of incident AF but there remain uncertainties regarding diabetes mellitus and the incidence of AF, in relation to type of diabetes, and the interactions with sex and age.

Patients with type II diabetes have a higher risk of prevalent AF (around 15%), and of incident AF (around 0.8%/year), which is partly related to other associated risk factors frequently encountered in these patients [1, 2]. Until recently, type I diabetes was considered as not being associated with incident AF, suggesting that long-term hyperglycaemia in diabetes per se would not itself promote AF; instead, the risk of AF seemed linked to insulin resistance, which is also the mechanism by which hypertension and obesity might be associated with an increased risk of AF [3, 4]. Obesity and type 2 diabetes are risk factors for AF [5], possibly because both increase epicardial adipose tissue, which is the source of proinflammatory adipocytokines that can lead to fibrosis of the underlying myocardium [6]. Diabetes could be an independent risk factor for AF when one specifically considers those patients aged < 75 [7]. Also, cardiovascular diseases are the major component of the excess risk of clinical events in diabetes and the proportional increase is higher among women than among men [8–10]. Compared with the general population, the risk of AF in men with type 1 diabetes is slightly raised, whereas for female patients it may be 50% higher [11]. An age and sex interaction with type of diabetes may thus influence sex-differences in the diabetes-related risk of AF. Moreover, several of the above findings are related to relatively older studies not taking into account contemporary changes in diabetes definition and management strategy [12, 13].

We further investigate these issues and performed a nationwide longitudinal cohort study to examine whether diabetes confers higher relative rates of AF in women compared to men, and whether these sex-differences depend on type of diabetes and age.

## Methods

### Study design

This longitudinal cohort study was based on the national hospitalization database covering hospital care from the entire French population. The data for all patients admitted in French hospitals in France from January to December 2013 with at least 5 years of complete follow-up (or dead earlier) were collected from the national administrative PMSI (*Programme de Médicalisation des Systèmes d’Information*) database, which was based on the US Medicare system. Through this program, medical activity is recorded in a database, computed, and rendered anonymous. It includes more than 98% of the French population (67 million people) from birth (or immigration) to death (or emigration), even if a person changes occupation or retires. This process allows the determination of each hospital’s budget, in 1546 French healthcare facilities for both public and private hospitals. Each hospitalization is encoded in a standardized dataset, which includes information about the patient (age during first hospitalization in 2013 and sex), hospital, stay (date of admission, date of discharge, and modes of discharge), pathologies, and procedures. Routinely collected medical information includes the principal diagnosis and secondary diagnoses. In the PMSI system, identified diagnoses are coded according to the International Classification of Diseases, Tenth Revision (ICD-10). All medical procedures are recorded according to the national nomenclature, Classification Commune des Actes Medicaux (CCAM). The PMSI contains individual anonymized information on each hospitalization that are linked to create a longitudinal record of hospital stays and diagnoses for each patient. The reliability of PMSI data has already been assessed and this database has previously been used to study patients with cardiovascular conditions, including AF [14–16]. Use of medication was identified from a 1/97 permanent random sample of the complete French nationwide claims database (Echantillon Généraliste de Bénéficiaires, EGB – general sample of healthcare beneficiaries), which is another database not linked to the PMSI database but has been previously used to study patients with diabetes in France [17]) We report information for patients with same inclusion criteria than those in the present analysis (patients seen in 2013 with at least 5 years of follow-up). Patients were considered to be included in a treatment group if they received a treatment from that class of drugs for ≥ 60 days within 6 months after enrolment.

The study was conducted retrospectively and, as patients were not involved in its conduct, there was no impact on their care. Ethical approval was not required, as all data were anonymized. The French Data Protection Authority granted access to the PMSI data. Procedures for data collection and management were approved by the Commission Nationale de l'Informatique et des Libertés (CNIL), the independent National Ethical Committee protecting human rights in France, which ensures that all information is kept confidential and anonymous, in compliance with the Declaration of Helsinki (authorization number 1897139).

### Study population

From 1 January 2013 to 31 December 2013, 3,381,472 adults (age ≥ 18 years) were hospitalized for any reason in French hospitals and then had at least 5 years of complete follow-up (or suffered in-hospital death earlier). Patient information (demographics, comorbidities, medical history, and events during hospitalization or follow-up) was described using data collected in the hospital records. For each hospital stay, combined diagnoses at discharge were obtained. Each diagnosis was identified using ICD-10 codes and because the information was on the basis of these codes, there were no missing values. Diabetes was identified with the following ICD-10 codes: E10, O240 for type 1 diabetes; E11, O241-9 for type 2 diabetes. Patients had a period of 3 years (2010 to 2013) to determine medical history. Exclusion criteria were age < 18 years and history of AF.

### Outcomes

Patients were followed until 31 December 2019 for the occurrence of outcomes. We aimed to evaluate the incidence of AF. This was evaluated with follow-up starting from the date of first hospitalization in 2013 until the date of AF or date of last news in the absence of the outcome. Information on AF during the follow-up was obtained by analysing the PMSI codes for each patient. We analysed the incidence of AF separately in patients with type 1 and type 2 diabetes, with non-diabetic patients as the reference group.

### Statistical analysis

Qualitative variables are described as frequency and percentages and quantitative variable as means (standard deviations [SDs]). A multivariable analysis for clinical outcomes during the whole follow-up in the groups of interests was performed using a Cox model with relevant baseline characteristics among age, cardiovascular risk factors (smoking, obesity, hypertension, dyslipidemia, alcohol related diagnoses) and non-cardiovascular comorbidities and reporting hazard ratios. We report incidence rates (IR = events/1000 person-years) and hazard ratios (HR) in women and in men, and then women-to-men ratios (WMR = HR women/HR men) with 95% confidence intervals (CIs). We present unadjusted WMR (unadjusted HR in women/unadjusted HR in men) and adjusted WMR (adjusted HR in women/adjusted HR in men).

Owing to the non-randomized nature of the study and considering the significant differences in baseline characteristics in women and men, propensity-score matching was also used to control for potential confounders. Propensity scores were calculated using logistic regression with female sex as the dependent variable. The propensity score included the cardiovascular risk factors and non-cardiovascular comorbidities from baseline characteristics listed in Table 1. For each female patient, a propensity score-matched male patient was selected (1:1) using the one-to-one nearest neighbour method (with a calliper of 0.0001 of the SD of the propensity score on the logit scale) and no replacement. We assessed the distributions of demographic data and comorbidities in the two cohorts with standardized differences, which were calculated as the difference in the means or proportions of a variable divided by a pooled estimate of the SD of that variable. A standardized difference of 5% or less indicated a marginal difference between means of the two cohorts (Additional file 1: Fig. S1).

**Table 1:** Baseline characteristics of patients seen in French hospitals in 2013 with at least 5 years of follow-up (mean follow-up 4.8 ± 1.7 years, median 5.5, IQR 5.1–5.8 years) according to sex and diabetes

	Women (n = 1,596,521)	Men (n = 1,324,886)	p	Total (n = 2,921,407)
Age, years	57.4 ± 20.2	62.4 ± 15.7	< 0.0001	59.7 ± 18.5
Hypertension	394,909 (24.7)	414,073 (31.3)	< 0.0001	808,982 (27.7)
Type 1 diabetes mellitus	21,440 (1.3)	23,949 (1.8)	< 0.0001	45,389 (1.6)
Type 2 diabetes mellitus	158,536 (9.9)	186,963 (14.1)	< 0.0001	345,499 (11.8)
Heart failure	78,953 (4.9)	118,967 (9.0)	< 0.0001	197,920 (6.8)
History of pulmonary edema	6134 (0.4)	8133 (0.6)	< 0.0001	14,267 (0.5)
Valve disease	31,293 (2.0)	33,494 (2.5)	< 0.0001	64,787 (2.2)
Aortic stenosis	12,777 (0.8)	15,286 (1.2)	< 0.0001	28,063 (1.0)
Aortic regurgitation	5961 (0.4)	6842 (0.5)	< 0.0001	12,803 (0.4)
Mitral regurgitation	11,861 (0.7)	11,598 (0.9)	< 0.0001	23,459 (0.8)
Previous endocarditis	719 (0.0)	1715 (0.1)	< 0.0001	2434 (0.1)
Dilated cardiomyopathy	15,679 (1.0)	25,281 (1.9)	< 0.0001	40,960 (1.4)
Coronary artery disease	80,028 (5.0)	180,628 (13.6)	< 0.0001	260,656 (8.9)
Previous myocardial infarction	13,781 (0.9)	29,458 (2.2)	< 0.0001	43,239 (1.5)
Previous PCI	17,071 (1.1)	54,082 (4.1)	< 0.0001	71,153 (2.4)
Previous CABG	1332 (0.1)	5455 (0.4)	< 0.0001	6787 (0.2)
Vascular disease	66,211 (4.1)	152,831 (11.5)	< 0.0001	219,042 (7.5)
Sinus node disease	4839 (0.3)	6677 (0.5)	< 0.0001	11,516 (0.4)
Previous pacemaker or ICD	18,282 (1.1)	35,437 (2.7)	< 0.0001	53,719 (1.8)
Ischemic stroke	18,064 (1.1)	24,153 (1.8)	< 0.0001	42,217 (1.4)
Intracranial bleeding	11,518 (0.7)	15,023 (1.1)	< 0.0001	26,541 (0.9)
Smoker	77,467 (4.9)	130,941 (9.9)	< 0.0001	208,408 (7.1)
Dyslipidemia	154,413 (9.7)	204,085 (15.4)	< 0.0001	358,498 (12.3)
Obesity	173,764 (10.9)	124,824 (9.4)	< 0.0001	298,588 (10.2)
Alcohol related diagnoses	43,979 (2.8)	123,782 (9.3)	< 0.0001	167,761 (5.7)
Chronic kidney disease	36,061 (2.3)	47,271 (3.6)	< 0.0001	83,332 (2.9)
Diabetic retinopathy	13,416 (0.8)	16,111 (1.2)	< 0.0001	29,527 (7.6)
Lung disease	113,325 (7.1)	146,430 (11.1)	< 0.0001	259,755 (8.9)
Sleep apnea syndrome	40,091 (2.5)	67,314 (5.1)	< 0.0001	107,405 (3.7)
COPD	46,967 (2.9)	92,916 (7.0)	< 0.0001	139,883 (4.8)
Liver disease	37,124 (2.3)	61,683 (4.7)	< 0.0001	98,807 (3.4)
Gastroesophageal reflux	55,429 (3.5)	43,668 (3.3)	< 0.0001	99,097 (3.4)
Thyroid diseases	111,988 (7.0)	27,696 (2.1)	< 0.0001	139,684 (4.8)
Inflammatory disease	84,357 (5.3)	66,951 (5.1)	< 0.0001	151,308 (5.2)
Anaemia	115,265 (7.2)	96,982 (7.3)	0.001	212,247 (7.3)
Previous cancer	197,346 (12.4)	245,236 (18.5)	< 0.0001	442,582 (15.1)
Poor nutrition	50,124 (3.1)	46,689 (3.5)	< 0.0001	96,813 (3.3)
Cognitive impairment	48,277 (3.0)	35,666 (2.7)	< 0.0001	83,943 (2.9)
Illicit drug use	4036 (0.3)	9041 (0.7)	< 0.0001	13,077 (0.4)

Values are n (%) or mean ± SD

*CABG* coronary artery bypass graft, *COPD* chronic obstructive pulmonary disease, *PCI* percutaneous coronary intervention, *SD* standard deviation

All comparisons with p < 0.05 were considered statistically significant. All analyses were performed using Enterprise Guide 7.1, (SAS Institute Inc., SAS Campus Drive, Cary, North Carolina), USA and STATA version 16.0 (Stata Corp, College Station, TX).

## Results

**Fig. 1:**
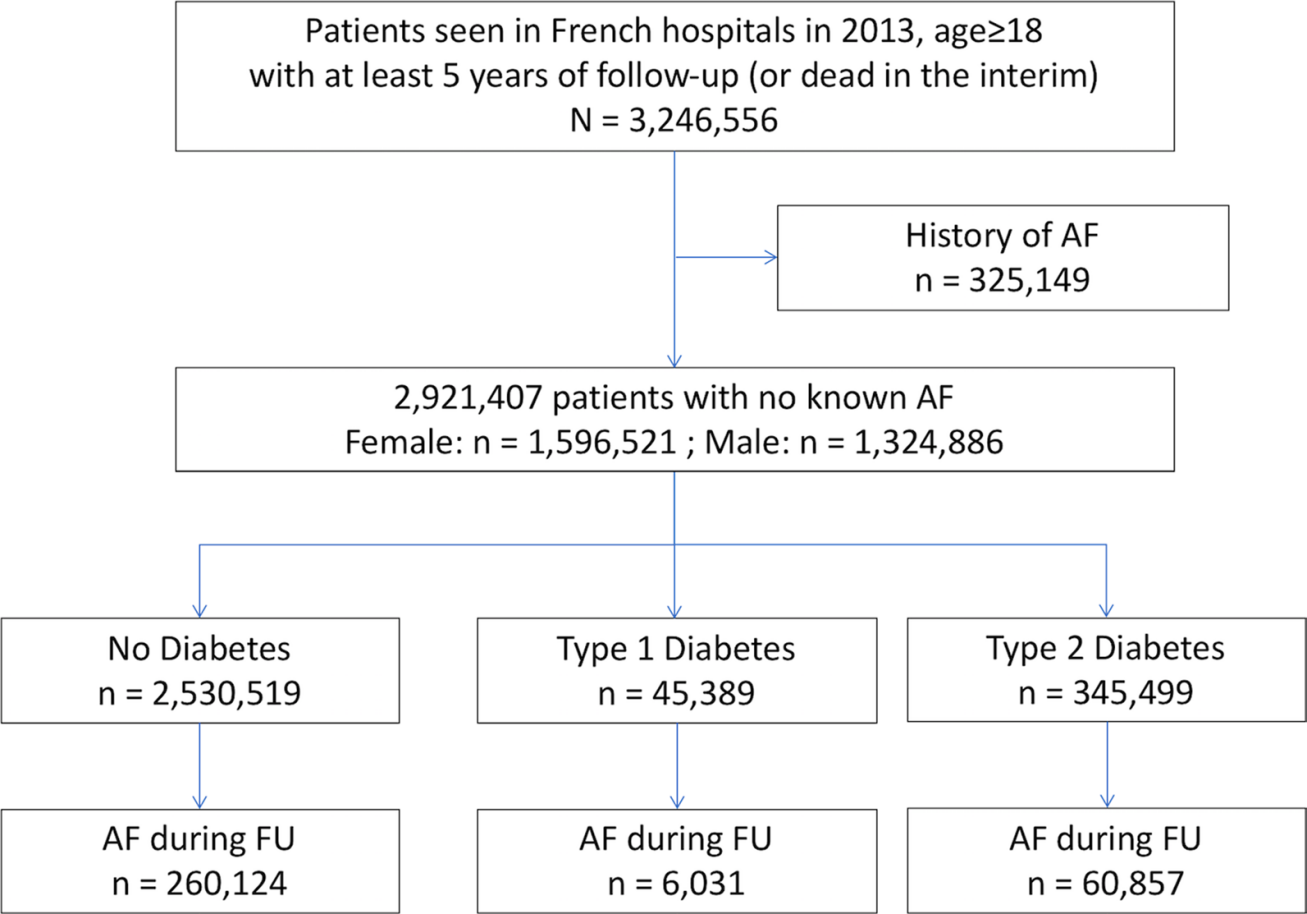
Flow chart of studied patients

We included 2,921,407 patients seen in French hospitals in 2013 with no history of AF (55% women), among whom 45,389 had prevalent type 1 diabetes and 345,499 had prevalent type 2 diabetes (Fig. 1). Characteristics of patients excluded because of AF are in Additional file 1: Table S1. Population characteristics at baseline showed that men were older and had more prevalent comorbidities than women (Table 1). Individuals with diabetes had more prevalent comorbidities than those without diabetes, and those with type 2 diabetes had more prevalent comorbidities than those with type 1 diabetes (Additional file 1: Table S2). Using data on medications for a 1/97 sample, those with diabetes were more frequently treated (approximately 2–threefold) with angiotensin-converting enzyme inhibitor or angiotensin receptor blocker, beta-blockers, diuretics, antiarrhythmic agents, anti-thrombotic therapy and statins than patients without diabetes (Additional file 1: Table S3). Among diabetic patients, women were less treated with these medications than men except for diuretics and calcium channel blocker.

**Fig. 2:**
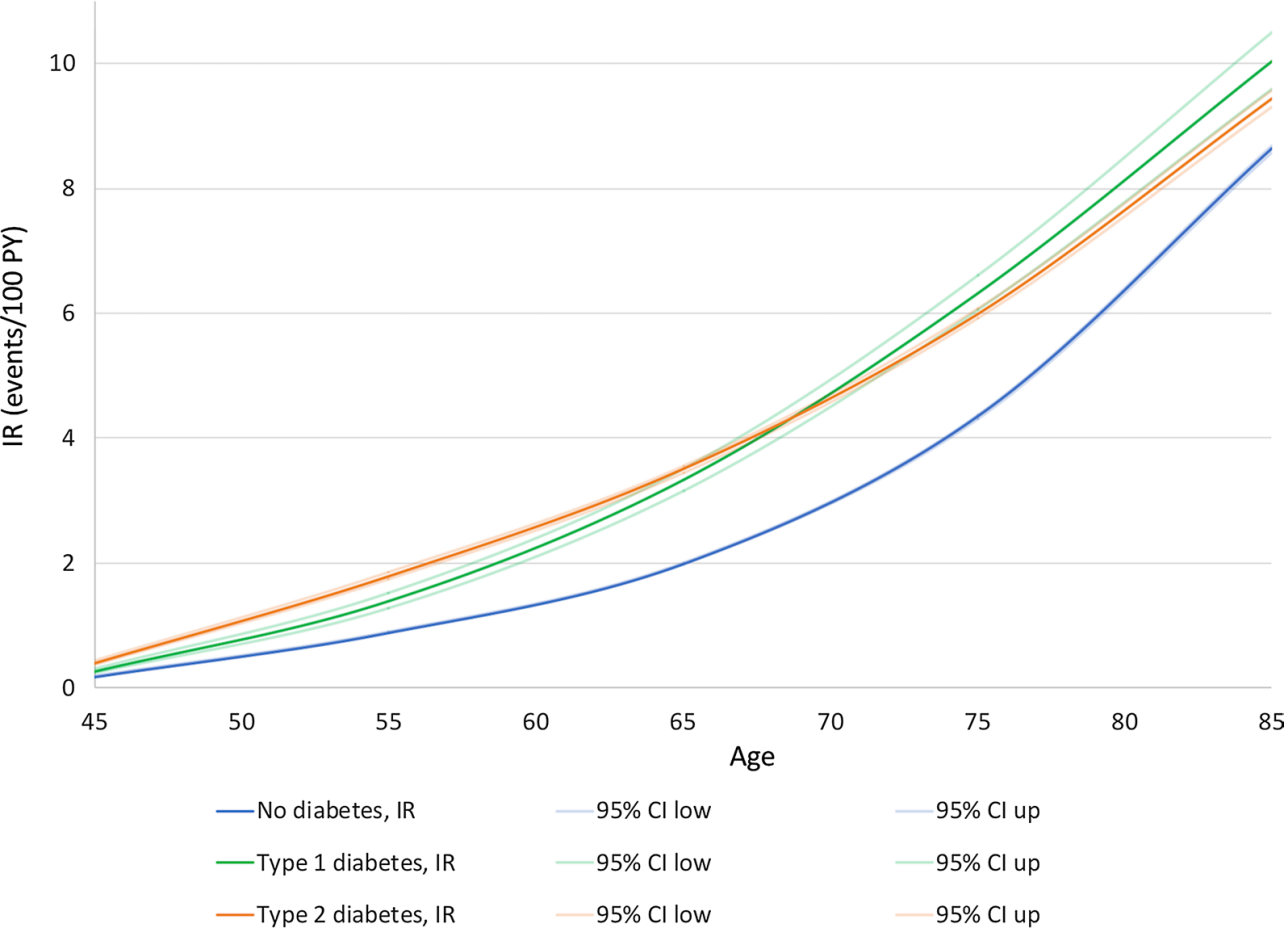
Incidence rates of atrial fibrillation according to age by decade, stratified by diabetes status

During 13.5 million person-years of follow-up, 327,012 patients with new-onset AF were identified. The IRs of AF was higher in diabetic than in non-diabetic patients and increased with advancing age, for both type 1 or type 2 diabetes (Fig. 2).

**Fig. 3:**
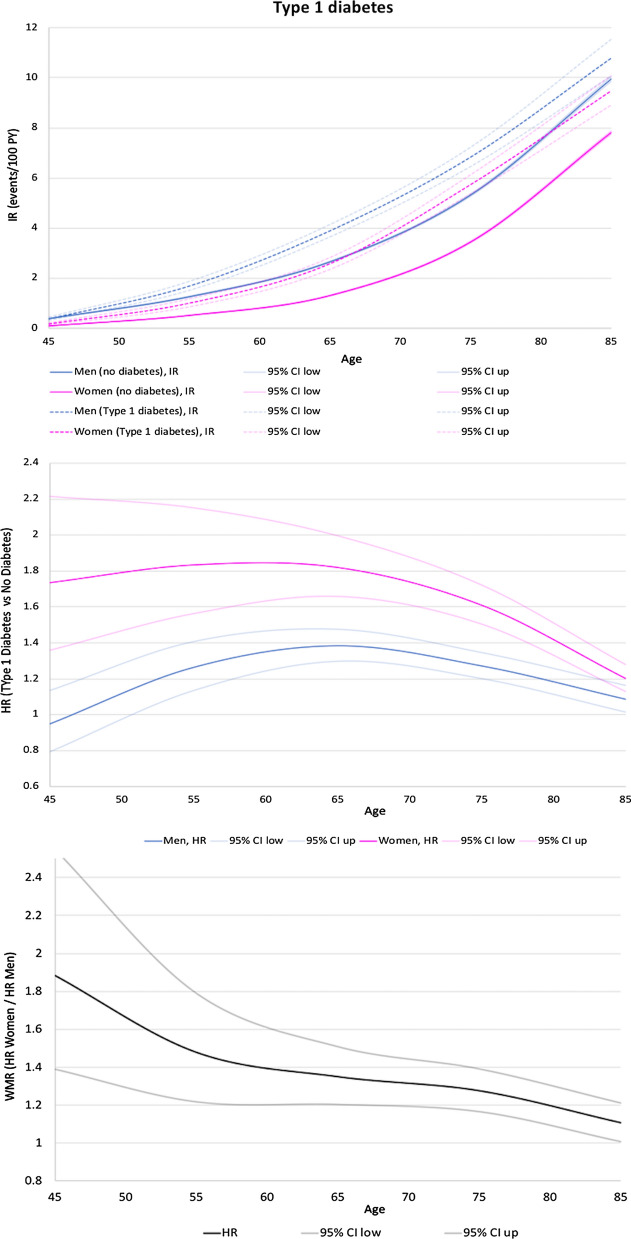
Incidence rates of first-time AF according to age, stratified by diabetes status and sex (top panel), hazard ratios stratified by sex (middle panel), and women-to-men ratios (lower panel) of first-time AF according to age by decade for type 1 diabetes vs no diabetes. Higher hazard ratio in women if women-to-men ratios > 1. HR, hazard ratios; WMR, women-to-men ratios

**Fig. 4:**
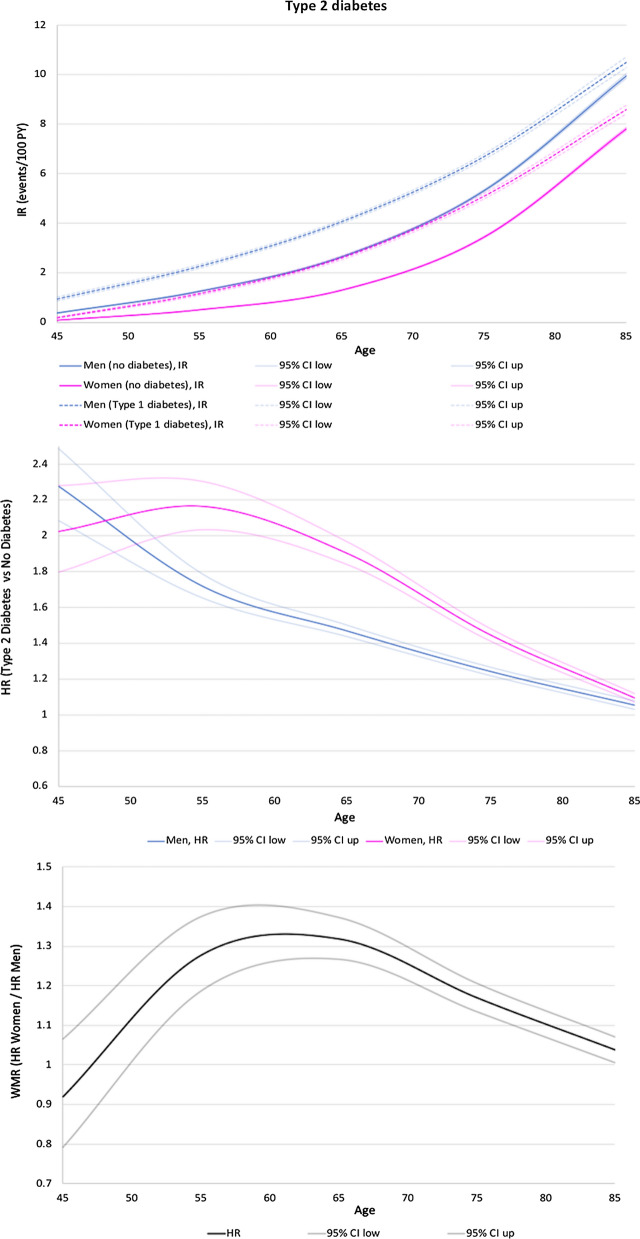
Incidence rates of first-time AF according to age, stratified by diabetes status and sex (top panel), hazard ratios stratified by sex (middle panel), and women-to-men ratios (lower panel) of first-time AF according to age by decade for type 2 diabetes vs no diabetes. Higher hazard ratio in women if women-to-men ratios > 1. HR, hazard ratios; WMR, women-to-men ratios

Among women, IR of AF was 1.68%/year in those with no diabetes, 2.63%/year in those with type 1 diabetes, and 3.38%/year in those with type 2 diabetes. The IRs for AF were higher in men than in women (2.92%/year in men with no diabetes, 3.37%/year in men with type 1 diabetes, and 4.96%/year in men with type 2 diabetes) irrespective of diabetes status and age (Table 2, Top panels in Fig. 3 and 4). The adjusted HR for women (diabetes:no diabetes) was therefore 1.32 (95% CI 1.27–1.37) for type 1 diabetes and 1.17 (95% CI 1.16–1.19) for type 2 diabetes. The corresponding adjusted HRs for men were 1.12 (95% CI 1.08–1.16) for type 1 diabetes and 1.10 (95% CI 1.09–1.12) for type 2 diabetes (Table 2). The adjusted HRs for women were significantly higher than the adjusted HRs for men as shown with the adjusted women-to-men ratios (adjusted WMR = adjusted HR women compared to adjusted HR men) = 1.18 (95% CI 1.12–1.24) for type 1 diabetes and 1.10 (95% CI 1.08–1.12) for type 2 diabetes (Table 2, Fig. 3 for type 1 diabetes and Fig. 4 for type 2 diabetes).

**Table 2:** Unadjusted and multivariable-adjusted HRs for incidence of AF comparing people with and without diabetes by sex, and hazard ratio for women relative to men

	Number	Person-time	Number of patients with AF during FU	Incidence rate (95% CI)	Hazard ratio (95% CI)		Women-to-men ratio (95% CI)		
					Diabetes vs no diabetes		(HR in women/HR in men)		
					Unadjusted	Adjusted	Unadjusted	Adjusted	
Women									
No diabetes	1,416,545	6,855,046.80	114 855	1.68 (1.67–1.69)					
Type 1 diabetes	21,440	97,121.95	2 557	2.63 (2.53–2.74)	1.50 (1.44–1.56)	1.32 (1.27–1.37)			
Type 2 diabetes	158,536	697,591.24	23,556	3.38 (3.33–3.42)	1.93 (1.90–1.96)	1.17 (1.16–1.19)			
							1.35 (1.28–1.42)	1.18 (1.12–1.24)	for type 1 diabetes
							1.20 (1.18–1.22)	1.10 (1.08–1.12)	for type 2 diabetes
Men									
No diabetes	1,113 974	4 971 106.10	145 269	2.92 (2.91–2.94)					
Type 1 diabetes	23,949	103,232.26	3 474	3.37 (3.26–3.48)	1.12 (1.09–1.16)	1.12 (1.08–1.16)			
Type 2 diabetes	186,963	751,998.44	37,301	4.96 (4.91–5.01)	1.64 (1.62–1.65)	1.10 (1.09–1.12)			

Adjusted for age at inclusion and baseline characteristics (among smoking, obesity, hypertension, hypercholesterolemia, alcohol abuse, previous stroke, and non-cardiovascular comorbidities). Women to men ratio > 1 indicates an excess risk for incident AF in women with prevalent type 1 or type 2 diabetes mellitus compared with men with prevalent similar type of diabetes mellitus

*HR* hazard ratio, *WMR* women-to-men ratios

This phenomenon was seen across all ages in men and women with type 1 diabetes and progressively decreased with advancing age. In type 2 diabetes, this phenomenon was seen after 50 years, increased until 60–65 years and then progressively decreased with advancing age (Fig. 3 and Fig. 4).

With propensity score matching, 1,107,874 female patients were adequately matched in a 1:1 fashion with male patients (mean age 62.4 ± 16.5, Additional file 1: Table S4, Fig. S1). Cumulative incidences for first-time AF in matched male and female patients during follow-up are presented in Additional file 1: Fig. S2, with lower incidence of AF in female patients. Additional file 1: Fig. S3 shows cumulative incidences for first-time AF during follow-up in matched male and female patients for type 1 diabetes and type 2 diabetes vs no diabetes. The overall results were consistent with those from the unmatched adjusted analysis. The higher risk of AF associated with type 1 diabetes was more marked in women than in men: HR 1.56, 95% CI 1.49–1.63 in women vs 1.12, 95% CI 1.08–1.16 in men, WMR (HR women compared to HR in matched men) 1.39, 95% CI 1.32–1.48. This difference was less marked with type 2 diabetes: HR 1.82, 95% CI 1.80–1.85 in women vs 1.67, 95% CI 1.65–1.69 in men, WMR 1.10, 95% CI 1.08–1.12.

## Discussion

In our large nationwide contemporary analysis our principal finding was that incidence of AF was significantly higher both in type 1 and type 2 diabetic patients than in patients with no diabetes, and both for men and for women. Second, there was a significantly higher relative risk of AF in diabetic versus nondiabetic women by comparison to the same risk evaluated in men, both for type 1 and type 2 diabetes. Third, we found that the sex differences across ages for the risk of AF were different for type 1 and type 2 diabetes.

In type 2 diabetes, AF is not uncommon, and for example, 7.6% of patients had AF at baseline in the ADVANCE study [18]. The incidence of AF has been reported to be around two-fold higher in patients with diabetes compared with people without diabetes [19], and AF incidence may be even higher in patients with microvascular complications (retinopathy, renal disease) [20]. In a meta-analysis which included 7 prospective studies and 4 case–control studies on 1.7 million subjects, diabetes was associated with about 40% higher risk of AF; however, after adjustment, the effect was more limited with an increased risk of only 24% [3]. The risk of type 2 diabetes on incident AF was 28% greater vs controls in a recent Swedish cohort study [21]. The results in our large adjusted analysis in almost 3 million subjects indicates that the adjusted excess risk was lower, around 15% (10% for men and 17% for women with type 2 diabetes). This was consistent with a previous analysis in 34,720 female health professionals which was inadequately powered to reach statistical significance (HR 1.14, 95% CI 0.93–1.40) [22]. Similarly, prevalence of elevated blood glucose level among residents in Guangzhou, China was associated with an increase in prevalence of AF, which was higher in women [23]. In a recent Spanish nationwide analysis, the incidence of hospitalization for AF was also higher in women with type 2 diabetes than in diabetic men [24]. Our original age-stratified analysis indicated that the excess risk in women was mainly observed in middle-aged patients. WMR increased after the age of 40 reaching a plateau at 1.3 at the age of 55 continuing until the age of 70 before sliding down to 1.1 at the age of 85.

Studies on type I diabetes in relation to AF risk are rarer, but recent large analyses eventually confirmed that type I diabetes was also independently associated with a higher incidence of AF [11, 25]. This point was previously a matter of debate, and (for example) was not reported in a recent analysis of 71,483 Swedish adults, which was underpowered considering the much lower prevalence of type 1 diabetes compared to type 2 diabetes [26]. By contrast, our analysis had sufficient statistical power to demonstrate that (1) type 1 diabetes was also associated with a significantly higher incidence of AF than in non-diabetics patients, indicating that insulin resistance would not be the only promoter of AF in patients with diabetes (2) this association was seen both in men and in women, (3) the adjusted hazard ratios for the risk of AF were actually even higher than for type 2 diabetes, particularly for women and (4) the higher relative risk of AF in women was seen across all ages, the women-to-men ratio being around 1.8 at age 45 and progressively decreasing with increasing age, which was somewhat different than what was seen for type 2. Recent pre-clinical studies suggested that loss of insulin signalling may contribute to atrial electrical remodelling and atrial fibrillation in murine models of type 1 diabetes [27].

Although several putative explanations may be suggested, there are currently no definite mechanisms for these sex differences across ages for type 1 and type 2 diabetes. These difference in risk may result from the complex interplay of hormonal homeostasis, other cardiovascular risk factors and psychosocial factors [28]. Diabetes may abrogate the protective effect of the female sex against cardiovascular complications across all age [29] and AF may be the consequence of these cardiovascular complications. Type 2 diabetes mellitus has been associated with impairment of nitric oxide dependent endothelial function in premenopausal diabetic women contributing to the incidence of some of these macrovascular complications [30]. Women with well-controlled diabetes mellitus may experience more coronary microvascular dysfunction leading to more severe diastolic function than diabetic men [31] which may also be a promoter for AF. High variability in bodyweight may also be associated with AF development in patients with type 2 diabetes, independently of traditional cardiovascular risk factors and baseline BMI, and is more likely to be seen in women [32]. Interestingly, the use of sodium-glucose cotransporter 2 (SGLT2) inhibitor drugs may provide protection against several cardiovascular adverse events, both for men and women with type 2 diabetes [33]. Among the SGLT2 inhibitor drugs, dapaglifozin has been shown to decrease the incidence of AF [34].

Nonetheless, these mechanisms may not fully explain the excess risk compared to men and other factors have been suggested such as less aggressive global control of risk factors [12, 35]. Women with diabetes may be less likely to have HbA1c < 7%, to receive lipid-lowering medication and, when treated, to achieve recommended lipid and blood pressure targets [36], whilst prevention of AF relies on the identification and management of risk factors and comorbidities predisposing to AF, before the development of atrial remodelling and fibrosis [37]. In our study, medication use was retrieved from a 1/97 representative sample of patients, and diabetic women were less likely to receive angiotensin-converting enzyme inhibitors, aspirin, P2Y12 inhibitors or statin than diabetic men. We acknowledge that this observation is only exploratory and therefore hypothesis generating. Our observations suggest that specific studies investigating more aggressive preventive measures in diabetic women are warranted. Of note, a higher rate of complications has been reported in women with catheter ablation of AF, which may be partially attributable to older age and a higher prevalence of comorbidities (including diabetes) at the time of ablation [38].

### Limitations

We acknowledge several limitations to our work. A main limitation is inherent to the retrospective, observational nature of the study and its potential biases. Further, the study was based on administrative data, with limitations inherent to such methodology. The PMSI database contains diagnoses coded using ICD-10, which are obtained at hospital discharge and are the physician’s responsibility. Data were not systematically externally checked and this could have caused information bias. However, the large scale of the database is likely to partly compensate some of these biases and, as coding of complications is linked to reimbursement and is regularly controlled, it is expected to be of good quality. Type 2 diabetes is widely treated in the primary sector by general practitioners and some of these patients may have not been included in the analysis. It is possible that patients with type 2 diabetes in this study could represent the more ill or with more comorbidity requiring in hospital care.

Events included were only in-hospital and we were not able to analyse data for out-of-hospital visits with AF. Our large population of hospitalized patients likely represents a heterogeneous group of patients admitted with various kinds of illnesses of different severities, which may have affected prognosis. Thus, the results might not be fully representative of more general population and further investigations would be valuable in the general ‘non-hospitalised’ population. Patients with diabetes had more prevalent comorbidities compared to those without diabetes and it is possible that those with diabetes were more often seen by healthcare personal, hence increasing the likelihood of silent AF being found randomly. However, there is no clear reason that this possible bias may more markedly affect women than men after adjustment on cardiovascular risk factors and non-cardiovascular comorbidities. Another limitation is the lack of complete information in terms of therapies recommended for diabetes or cardiovascular conditions beyond the representative sample from our analysis. Further, the non-randomized design of the analysis leaves a risk of residual confounding factors. Our analysis was restricted to the variables present in the database, which meant that characteristics such as information on some of the lifestyle factors (physical activity level or diet), metabolic control (glycemic control, lipids, body mass index and blood pressure) or imaging (echocardiography including measures of systolic function or left atrial size) were not available for analysis. Finally, the majority of the French population is white European and our results may not be generalizable to non-white European people.

## Conclusions

In a contemporary nationwide study, we found that diabetes was associated with a 10% to 30% higher incidence of AF after adjustment on other baseline risk factors, which was highest in women with type 1 diabetes. Although diabetic men have higher absolute incidence of AF, the relative incidences of AF were higher in diabetic women than in diabetic men, both for type 1 and type 2 diabetes. This phenomenon was seen across all ages in men and women with type 1 diabetes and progressively decreased with advancing age. By contrast, the excess risk in women compared to men was mainly observed in middle-aged patients in type 2 diabetes.

## Supplementary Information

**Additional file 1.** Additional figures and tables.

## Data Availability

Because this study used data from human subjects, the data and everything pertaining to the data are governed by the French Health Agencies and cannot be made available to other researchers.

## Abbreviations

AF: Atrial fibrillation
CCAM: Classification Commune des Actes Medicaux
CI: Confidence interval
CNIL: Commission Nationale de l'Informatique et des Libertés
EGB: Echantillon Généraliste de Bénéficiaires
HR: Hazard ratio
ICD-10: International Classification of Diseases, Tenth Revision
IR: Incidence rate
PMSI: Programme de Médicalisation des Systèmes d’Information
WMR: Women-to-men ratios
