# Supplementary material for: Sex, age, type of diabetes and incidence of atrial fibrillation in patients with diabetes mellitus: a nationwide analysis
Source: Cardiovasc Diabetol. 2021 Jan 22;20:24. doi: 10.1186/s12933-021-01216-7 (PMC7821402; doi:10.1186/s12933-021-01216-7)
Supplement: Supplementary file 1 — Additional file 1. Additional figures and tables. [file 12933_2021_1216_MOESM1_ESM.docx]

**Sex, age, type of diabetes and incidence of atrial fibrillation in patients with diabetes mellitus: a nationwide analysis. *Additional table and figures***

**Additional file 1: Table S1.** Baseline characteristics of patients seen in French hospitals in 2013 included (no history of atrial fibrillation) or excluded from the analysis (history of atrial fibrillation).

|  | **No Atrial fibrillation** | **Atrial fibrillation** | **p** | **Total** |
| --- | --- | --- | --- | --- |
|  | **(n=2921407)** | **(n=325149)** |  | **(n=3246556)** |
| **Age, years** | 59.7±18.5 | 78.0±10.6 | <0.0001 | 61.5±18.7 |
| **Sex (male)** | 1324886 (45.4) | 183569 (56.5) | <0.0001 | 1508455 (46.5) |
| **Hypertension** | 808982 (27.7) | 224271 (69.0) | <0.0001 | 1033253 (31.8) |
| **Type 1 diabetes mellitus** | 45389 (1.6) | 5234 (1.6) | 0.01 | 50623 (1.6) |
| **Type 2 diabetes mellitus** | 345499 (11.8) | 79708 (24.5) | <0.0001 | 425207 (13.1) |
| **Heart failure** | 197920 (6.8) | 156519 (48.1) | <0.0001 | 354439 (10.9) |
| **History of pulmonary edema** | 14267 (0.5) | 11532 (3.5) | <0.0001 | 25799 (0.8) |
| **Valve disease** | 64787 (2.2) | 56757 (17.5) | <0.0001 | 121544 (3.7) |
| **Aortic stenosis** | 28063 (1.0) | 23281 (7.2) | <0.0001 | 51344 (1.6) |
| **Aortic regurgitation** | 12803 (0.4) | 10401 (3.2) | <0.0001 | 23204 (0.7) |
| **Mitral regurgitation** | 23459 (0.8) | 26972 (8.3) | <0.0001 | 50431 (1.6) |
| **Previous endocarditis** | 2434 (0.1) | 2106 (0.6) | <0.0001 | 4540 (0.1) |
| **Dilated cardiomyopathy** | 40960 (1.4) | 37036 (11.4) | <0.0001 | 77996 (2.4) |
| **Coronary artery disease** | 260656 (8.9) | 100712 (31.0) | <0.0001 | 361368 (11.1) |
| **Previous myocardial infarction** | 43239 (1.5) | 14465 (4.4) | <0.0001 | 57704 (1.8) |
| **Previous PCI** | 71153 (2.4) | 18111 (5.6) | <0.0001 | 89264 (2.7) |
| **Previous CABG** | 6787 (0.2) | 5530 (1.7) | <0.0001 | 12317 (0.4) |
| **Vascular disease** | 219042 (7.5) | 72620 (22.3) | <0.0001 | 291662 (9.0) |
| **Sinus node disease** | 11516 (0.4) | 13258 (4.1) | <0.0001 | 24774 (0.8) |
| **Previous pacemaker or ICD** | 53719 (1.8) | 51286 (15.8) | <0.0001 | 105005 (3.2) |
| **Ischemic stroke** | 42217 (1.4) | 21690 (6.7) | <0.0001 | 63907 (2.0) |
| **Intracranial bleeding** | 26541 (0.9) | 8345 (2.6) | <0.0001 | 34886 (1.1) |
| **Smoker** | 208408 (7.1) | 23837 (7.3) | <0.0001 | 232245 (7.2) |
| **Dyslipidemia** | 358498 (12.3) | 86791 (26.7) | <0.0001 | 445289 (13.7) |
| **Obesity** | 298588 (10.2) | 58765 (18.1) | <0.0001 | 357353 (11.0) |
| **Alcohol related diagnoses** | 167761 (5.7) | 20389 (6.3) | <0.0001 | 188150 (5.8) |
| **Chronic kidney disease** | 83332 (2.9) | 36083 (11.1) | <0.0001 | 119415 (3.7) |
| **Diabetic retinopathy** | 29741 (1.0) | 4568 (1.4) | <0.0001 | 34309 (1.1) |
| **Lung disease** | 259755 (8.9) | 76484 (23.5) | <0.0001 | 336239 (10.4) |
| **Sleep apnea syndrome** | 107405 (3.7) | 26711 (8.2) | <0.0001 | 134116 (4.1) |
| **COPD** | 139883 (4.8) | 47458 (14.6) | <0.0001 | 187341 (5.8) |
| **Liver disease** | 98807 (3.4) | 16090 (4.9) | <0.0001 | 114897 (3.5) |
| **Gastroesophageal reflux** | 99097 (3.4) | 7295 (2.2) | <0.0001 | 106392 (3.3) |
| **Thyroid diseases** | 139684 (4.8) | 44102 (13.6) | <0.0001 | 183786 (5.7) |
| **Inflammatory disease** | 151308 (5.2) | 25149 (7.7) | <0.0001 | 176457 (5.4) |
| **Anaemia** | 212247 (7.3) | 62766 (19.3) | <0.0001 | 275013 (8.5) |
| **Previous cancer** | 442582 (15.1) | 61172 (18.8) | <0.0001 | 503754 (15.5) |
| **Poor nutrition** | 96813 (3.3) | 31368 (9.6) | <0.0001 | 128181 (3.9) |
| **Cognitive impairment** | 83943 (2.9) | 30859 (9.5) | <0.0001 | 114802 (3.5) |
| **Illicit drug use** | 13077 (0.4) | 435 (0.1) | <0.0001 | 13512 (0.4) |

Values are n (%) or mean±SD. CABG = coronary artery bypass graft; COPD = chronic obstructive pulmonary disease; PCI = percutaneous coronary intervention; SD = standard deviation.

**Additional file 1: Table S2.** Baseline characteristics of patients seen in French hospitals in 2013 with at least 5 years of follow-up according to type of diabetes.

|  | **No diabetes** | **Type 1 diabetes** | **Type 2 diabetes** | **p for diabetes vs no diabetes** | **p for type 1**  **vs type 2 diabetes** |
| --- | --- | --- | --- | --- | --- |
|  | **(n=2530519)** | **(n=45389)** | **(n=345499)** |  |  |
| **Age, years** | 58.7±18.8 | 59.6±18.4 | 66.7±14.5 | <0.0001 | <0.0001 |
| **Gender (male)** | 1113974 (44.0) | 23949 (52.8) | 186963 (54.1) | <0.0001 | <0.0001 |
| **Hypertension** | 553728 (21.9) | 21677 (47.8) | 233577 (67.6) | <0.0001 | <0.0001 |
| **Heart failure** | 129972 (5.1) | 5458 (12.0) | 62490 (18.1) | <0.0001 | <0.0001 |
| **History of pulmonary edema** | 9768 (0.4) | 461 (1.0) | 4038 (1.2) | <0.0001 | 0.004 |
| **Valve disease** | 47934 (1.9) | 1080 (2.4) | 15773 (4.6) | <0.0001 | <0.0001 |
| **Aortic stenosis** | 19832 (0.8) | 499 (1.1) | 7732 (2.2) | <0.0001 | <0.0001 |
| **Aortic regurgitation** | 9899 (0.4) | 139 (0.3) | 2765 (0.8) | <0.0001 | <0.0001 |
| **Mitral regurgitation** | 17378 (0.7) | 370 (0.8) | 5711 (1.7) | <0.0001 | <0.0001 |
| **Previous endocarditis** | 1776 (0.1) | 46 (0.1) | 612 (0.2) | <0.0001 | 0.0002 |
| **Dilated cardiomyopathy** | 28032 (1.1) | 833 (1.8) | 12095 (3.5) | <0.0001 | <0.0001 |
| **Coronary artery disease** | 171339 (6.8) | 7463 (16.4) | 81854 (23.7) | <0.0001 | <0.0001 |
| **Previous myocardial infarction** | 30365 (1.2) | 963 (2.1) | 11911 (3.4) | <0.0001 | <0.0001 |
| **Previous PCI** | 47300 (1.9) | 1588 (3.5) | 22265 (6.4) | <0.0001 | <0.0001 |
| **Previous CABG** | 3811 (0.2) | 193 (0.4) | 2783 (0.8) | <0.0001 | <0.0001 |
| **Vascular disease** | 144473 (5.7) | 6415 (14.1) | 68154 (19.7) | <0.0001 | <0.0001 |
| **Sinus node disease** | 8605 (0.3) | 185 (0.4) | 2726 (0.8) | <0.0001 | <0.0001 |
| **Previous pacemaker or ICD** | 39153 (1.5) | 1133 (2.5) | 13433 (3.9) | <0.0001 | <0.0001 |
| **Ischemic stroke** | 30947 (1.2) | 809 (1.8) | 10461 (3.0) | <0.0001 | <0.0001 |
| **Intracranial bleeding** | 22135 (0.9) | 375 (0.8) | 4031 (1.2) | <0.0001 | <0.0001 |
| **Smoker** | 168115 (6.6) | 4529 (10.0) | 35764 (10.4) | <0.0001 | 0.01 |
| **Dyslipidemia** | 221998 (8.8) | 10415 (22.9) | 126085 (36.5) | <0.0001 | <0.0001 |
| **Obesity** | 180030 (7.1) | 8117 (17.9) | 110441 (32.0) | <0.0001 | <0.0001 |
| **Alcohol related diagnoses** | 136864 (5.4) | 3405 (7.5) | 27492 (8.0) | <0.0001 | 0.001 |
| **Chronic kidney disease** | 50405 (2.0) | 3787 (8.3) | 29140 (8.4) | <0.0001 | 0.51 |
| **Diabetic retinopathy** | 0 (0.0) | 5934 (13.1) | 23593 (6.8) | <0.0001 | <0.0001 |
| **Lung disease** | 201710 (8.0) | 4608 (10.2) | 53437 (15.5) | <0.0001 | <0.0001 |
| **Sleep apnea syndrome** | 69520 (2.7) | 2400 (5.3) | 35485 (10.3) | <0.0001 | <0.0001 |
| **COPD** | 106168 (4.2) | 2313 (5.1) | 31402 (9.1) | <0.0001 | <0.0001 |
| **Liver disease** | 67760 (2.7) | 2745 (6.0) | 28302 (8.2) | <0.0001 | <0.0001 |
| **Gastroesophageal reflux** | 87673 (3.5) | 911 (2.0) | 10513 (3.0) | <0.0001 | <0.0001 |
| **Thyroid diseases** | 104315 (4.1) | 4121 (9.1) | 31248 (9.0) | <0.0001 | 0.81 |
| **Inflammatory disease** | 127399 (5.0) | 2079 (4.6) | 21830 (6.3) | <0.0001 | <0.0001 |
| **Anaemia** | 159690 (6.3) | 4910 (10.8) | 47647 (13.8) | <0.0001 | <0.0001 |
| **Previous cancer** | 378930 (15.0) | 5824 (12.8) | 57828 (16.7) | <0.0001 | <0.0001 |
| **Poor nutrition** | 75561 (3.0) | 2334 (5.1) | 18918 (5.5) | <0.0001 | 0.003 |
| **Cognitive impairment** | 65882 (2.6) | 1414 (3.1) | 16647 (4.8) | <0.0001 | <0.0001 |
| **Illicit drug use** | 11905 (0.5) | 258 (0.6) | 914 (0.3) | <0.0001 | <0.0001 |

Values are n (%) or mean±SD. CABG = coronary artery bypass graft; COPD = chronic obstructive pulmonary disease; PCI = percutaneous coronary intervention; SD = standard deviation.

**Additional file 1: Table S3.** Rate of medication at discharge after a first hospitalization in 2013 for patients with diabetes and no diabetes.

|  | **Women** | | **Men** | |
| --- | --- | --- | --- | --- |
|  | **No Diabetes** | **Diabetes** | **No Diabetes** | **Diabetes** |
|  | (n= 19,828) | (n= 2,440) | (n= 13,966) | (n= 2,907) |
| Age, years | 52.4 ± 20.6 | 65.6 ± 15.2 | 56.8 ± 17.6 | 65.6 ± 12.4 |
| ACE inhibitor or ARB | 3,248 (16.4%) | 1,296 (53.1%) | 3,333 (23.9%) | 1,703 (58.6%) |
| Beta-blocker | 2,358 (11.9%) | 773 (31.7%) | 2,071 (14.8%) | 994 (34.2%) |
| Diuretic | 1,589 (8.0%) | 604 (24.8%) | 1,057 (7.6%) | 631 (21.7%) |
| K-sparing diuretics | 320 (1.6%) | 93 (3.8%) | 220 (1.6%) | 131 (4.5%) |
| Calcium channel blocker | 1,429 (7.2%) | 550 (22.5%) | 1,340 (9.6%) | 674 (23.2%) |
| Antiarrhythmic agents | 356 (1.8%) | 56 (2.3%) | 332 (2.4%) | 92 (3.2%) |
| Amiodarone | 155 (0.8%) | 27 (1.1%) | 183 (1.3%) | 65 (2.2%) |
| VKA | 543 (2.7%) | 112 (4.6%) | 553 (4.0%) | 176 (6.1%) |
| Direct oral anticoagulant | 72 (0.4%) | 12 (0.5%) | 59 (0.4%) | 19 (0.7%) |
| Aspirin | 1,649 (8.3%) | 678 (27.8%) | 2,058 (14.7%) | 1,048 (36.1%) |
| P2Y12 inhibitor | 440 (2.2%) | 222 (9.1%) | 917 (6.6%) | 499 (17.2%) |
| Statin | 2,330 (11.8%) | 1,032 (42.3%) | 2,798 (20.0%) | 1,459 (50.2%) |
| Antidiabetic | - | 2,099 (86.0%) | - | 2,513 (86.4%) |
| Metformin | - | 925 (37.9%) | - | 1,202 (41.3%) |
| Insulin | - | 802 (32.9%) | - | 807 (27.8%) |
| Sulfonylureas | - | 607 (24.9%) | - | 820 (28.2%) |
| GLP1-analogues | - | 91 (3.7%) | - | 98 (3.4%) |
| DPP4-inhibitors | - | 277 (11.4%) | - | 338 (11.6%) |

% of use for each medication was identified in the EGB permanent random sample (1/97) of the French nationwide claims database (n= 39,141patients seen in 2013, age ≥18, no history of AF with at least 5 years of follow-up). ACE, angiotensin-converting enzyme; ARB, angiotensin receptor blocker. DPP4, dipeptidyl peptidase 4; GLP1, glucagon-like peptide 1. VKA, Vitamin K-antagonists.

**Additional file 1: Table S4.** Baseline characteristics of matched male and female patients seen in French hospitals in 2013 with at least 5 years of follow-up.

|  | **Women** | **Men** | **Standardized difference,** | | **Total** | |
| --- | --- | --- | --- | --- | --- | --- |
|  | **(n=1107874)** | **(n=1107874)** | **(%)** | | **(n=2215748)** | |
| **Age, years** | 62.7±16.7 | 62.1±16.3 | 3.7 | | 62.4±16.5 | |
| **Hypertension** | 312397 (28.2) | 305532 (27.6) | 1.4 | | 617929 (27.9) | |
| **Type 1 diabetes mellitus** | 14388 (1.3) | 19344 (1.7) | - | 33732 (1.5) | |  |
| **Type 2 diabetes mellitus** | 115958 (10.5) | 139081 (12.6) | - | 255039 (11.5) | |  |
| **Ischemic stroke** | 15610 (1.4) | 16239 (1.5) | -0.5 | 31849 (1.4) | |  |
| **Intracranial bleeding** | 10479 (0.9) | 10672 (1.0) | -0.2 | 21151 (1.0) | |  |
| **Smoker** | 60309 (5.4) | 63219 (5.7) | -1.0 | 123528 (5.6) | |  |
| **Dyslipidemia** | 128637 (11.6) | 131217 (11.8) | -0.7 | 259854 (11.7) | |  |
| **Obesity** | 111199 (10.0) | 100546 (9.1) | 3.2 | 211745 (9.6) | |  |
| **Alcohol related diagnoses** | 43370 (3.9) | 45129 (4.1) | -0.7 | 88499 (4.0) | |  |
| **Chronic kidney disease** | 30976 (2.8) | 32336 (2.9) | -0.7 | 63312 (2.9) | |  |
| **Lung disease** | 87520 (7.9) | 88421 (8.0) | -0.3 | 175941 (7.9) | |  |
| **Sleep apnea syndrome** | 36931 (3.3) | 34896 (3.1) | 1.0 | 71827 (3.2) | |  |
| **COPD** | 44322 (4.0) | 44019 (4.0) | 0.1 | 88341 (4.0) | |  |
| **Liver disease** | 32562 (2.9) | 31722 (2.9) | 0.4 | 64284 (2.9) | |  |
| **Gastroesophageal reflux** | 42344 (3.8) | 38036 (3.4) | 2.2 | 80380 (3.6) | |  |
| **Thyroid diseases** | 29544 (2.7) | 27031 (2.4) | 1.1 | 56575 (2.6) | |  |
| **Inflammatory disease** | 46333 (4.2) | 56806 (5.1) | -4.3 | 103139 (4.7) | |  |
| **Anaemia** | 75895 (6.9) | 76180 (6.9) | -0.1 | 152075 (6.9) | |  |
| **Previous cancer** | 174645 (15.8) | 173044 (15.6) | 0.4 | 347689 (15.7) | |  |
| **Poor nutrition** | 36145 (3.3) | 34731 (3.1) | 0.7 | 70876 (3.2) | |  |
| **Cognitive impairment** | 32709 (3.0) | 30814 (2.8) | 1.0 | 63523 (2.9) | |  |
| **Illicit drug use** | 3885 (0.4) | 4004 (0.4) | -0.2 | 7889 (0.4) | |  |

Values are n (%) or mean±SD. CABG = coronary artery bypass graft; COPD = chronic obstructive pulmonary disease; PCI = percutaneous coronary intervention; SD = standard deviation. Matching for age at inclusion and baseline characteristics (among smoking, obesity, hypertension, hypercholesterolemia, alcohol abuse, previous stroke, and non-cardiovascular comorbidities).

**Additional file 1: Figure S1.** Standardized percentages of bias across main baseline characteristics in unmatched and matched male and female patients.

**Additional file 1: Figure S2.** Cumulative incidences for first-time AF in matched male and female patients during follow-up.

**Additional file 1: Figure S3.** Cumulative incidences for first-time AF during follow-up in matched male and female patients for type 1 diabetes vs no diabetes (top panel) and type 2 diabetes vs no diabetes (lower panel).
